# Determination of Zinc Status in Humans: Which Indicator Should We Use?

**DOI:** 10.3390/nu7053252

**Published:** 2015-05-06

**Authors:** Frank T. Wieringa, Marjoleine A. Dijkhuizen, Marion Fiorentino, Arnauld Laillou, Jacques Berger

**Affiliations:** 1 Institute of Research for Development (IRD), UMR Nutripass IRD-UM2-UM1, 911 Avenue d’Agropolis, 34394 Montpellier, France; E-Mails: marionfiorentino@hotmail.com (M.F.); Jacques.Berger@ird.fr (J.B.); 2 Department of Nutrition, Exercise and Sports (NEXS), Copenhagen University, Rolighedsvej 26, 1958 Frederiksberg, Denmark; E-Mail: madijkhuizen@gmail.com; 3 UNICEF, Maternal Child Health and Nutrition Section, No.11 street 75, 12200 Phnom Penh, Cambodia; E-Mail: alaillou@unicef.org

**Keywords:** zinc, biomarkers, stunting, anemia, dietary consumption, South East Asia, Vietnam

## Abstract

Zinc deficiency has serious wide-ranging health consequences and is thought to be one of the most prevalent micronutrient deficiencies in the world. However, reliable indicators or biomarkers to assess zinc status are not available at present. Indirect indicators such as the prevalence of stunting or anemia, iron deficiency, as well as more direct indicators such as plasma zinc concentrations are being used at present to estimate the prevalence of zinc deficiency in populations. However, as this paper shows by using data from a recent national micronutrient survey in Vietnam, the estimates of the prevalence of zinc deficiency using these different indicators can vary widely, leading to inconsistencies. In this paper, zinc deficiency among children is four times more prevalent than iron deficiency and 2.3 times more than stunting prevalence for example. This can lead not only to confusion concerning the real extent of the prevalence of zinc deficiency in populations, but also makes it hard to inform policy on whether action is needed or not. Moreover, evaluation of programs is hampered by the lack of a clear indicator. Efforts should be made to identify the most suitable indicator to evaluate the impact of programs aimed at improving zinc status and health of populations.

## 1. Introduction

Zinc is an essential mineral for human health. It is relatively abundant in nature, yet at the same time, overwhelming data suggest that zinc deficiency is one of the most prevalent micronutrient deficiencies worldwide [1]. Already in the 1930s it was shown that zinc was essential for normal growth in rodents [2], but it was only realized in 1961 that zinc deficiency in humans was actually causing a syndrome called “adolescent nutritional dwarfism” [2,3]. Nowadays, it is recognized that zinc is important for many basic metabolic processes, and hence essential for optimal growth, immunocompetence, and even visual acuity. Indeed, zinc deficiency has been linked to ~116,000 child deaths each year, mainly through increased prevalence and severity of infectious diseases such as diarrhea [4].

One important reason that it took so long before zinc was recognized as an important mineral for public health in humans was the lack of a reliable indicator for zinc status. Most micronutrient deficiencies are associated with a clear clinical syndrome and/or a unique biomarker. Often, the biomarker is directly associated with negative health outcomes. For example, severe vitamin A deficiency is characterized by the clinical syndrome xerophthalmia which is strongly associated with low plasma retinol concentrations (biomarker) [5]. Thus, the prevalence of vitamin A deficiency, measured either as prevalence of xerophtalmia or the prevalence of subjects with plasma retinol concentrations below a certain cut-off, can serve as indicator for the severity of the deficiency in a population. Importantly, these indicators can guide policy, both in developing health programs and in monitoring and evaluation of the impact of interventions.

In some instances the association between status and indicator (either the clinical syndrome or the biomarker) might not be so straightforward, thereby making the indicator less useful, and the health implications of deficiency less easily recognizable. For instance, although iron deficiency is for most people directly related to anemia, it must be realized that anemia can be caused by many other factors, and the exact role of iron in health and disease is more complex than previously thought. Therefore, iron deficiency should not be considered equal to anemia. However, anemia is still a useful indicator for iron deficiency, especially when combined with other secondary biomarkers [6].

For zinc deficiency, however, we lack a clear indicator, as there is no specific syndrome or biomarker linked to zinc deficiency. Zinc deficiency usually leads to very general signs or symptoms such as growth impairment and general immune impairment, but these symptoms can result from many other causes and hence do not specifically point to zinc deficiency. Maybe as a result of this lack of clear signs or specific symptoms for zinc deficiency, many people have tried to come up with suitable biomarkers for zinc status. For a complete overview of biomarkers used to determine zinc status, a recent review by Lowe *et al.* (2009) identified 32 (!) biomarkers for zinc status [7]. However, most of these biomarkers are seldom used for assessment of zinc status in populations, with the exception of zinc concentrations in plasma (or serum). Indeed, of the papers found by the search of Lowe and colleagues, only plasma zinc concentrations had >50 references, whereas all other indicators had less than 10 references, and most even only 1. Worrisome, Lowe *et al.* [7] identified only 3 out of 32 biomarkers as useful (plasma, urine and hair zinc concentrations). Although interesting from a scientific point of view, policymakers will be hard to convince by results of exotic biomarkers, or markers which do not provide information on zinc status at a population level. The current paper will therefore discuss the limitations of some of the most widely used biomarkers for population surveys, and use data from a recent National Micronutrient Survey in Vietnam to highlight the policy implications of this lack of a reliable indicator for zinc deficiency [8].

## 2. Methods

### 2.1. Study Design and Sampling

In 2009, a nationwide food consumption survey was carried in Vietnam among young children and women of reproductive age from 7680 households randomly selected using a stratified 2-stage cluster sampling procedure with probability proportionate to size (104 urban and 408 rural clusters). In 2010, a part of these participants were re-surveyed to provide additional information on micronutrient status of women in reproductive age and young children. The 2010 micronutrient study was carried-out in a subset of 840 same households from 56 urban and 56 rural clusters (15 households per cluster) from 19 randomly selected provinces.

Selected women and children were invited to come to the Commune Health Center early morning. Children and women were weighed without shoes or sandals and wearing light clothes by using a balance with a precision of 0.1 kg (Body Composition Monitor Scale Tanita BC-543, Tokyo, Japan). Height was measured by using a height measuring device with a precision of 0.1 cm (wooden height board, UNICEF). Anthropometric z-scores were calculated using the National Center for Health Statistics/WHO growth reference data of 2006 [9]. For women, body mass index (BMI) was calculated as the body weight (in kg) divided by the square of height (in meters). Women were classified using the BMI cut-off points endorsed for Asian populations: underweight (BMI < 18.5 kg/m^2^), healthy weight (BMI 18.5–22.9 kg/m^2^), overweight (BMI 23.0–27.49 kg/m^2^), and obese (BMI ≥ 27.5 kg/m^2^) [10].

After anthropometry, a non-fasting blood sample was collected by venipuncture in trace-element free heparinized sampling tubes (Vacuette, Greiner Bio One, Kremsmünster, Austria). Plasma was aliquoted into 200 μL pre-labeled Eppendorf tubes and kept frozen at −20 °C at the Provincial Health Centre before being sent (within two weeks) on dry ice to the National Institute of Nutrition (NIN) where samples were stored at −70 °C until analysis.

Food intakes of children and women included in this study were extracted from the food consumption data at the household level and the individual consumption of children aged less than 5 years old measured during the 2009 Food Consumption Survey (FCS) using the 24-h recall method combined with controlled food weighing [11]. The 24-h recall was conducted by teams of dietitians or trained personnel from the provincial medical centers. For children under 5 years of age, a specific questionnaire was implemented to estimate, through the 24-h recall, the child consumption. The dietitians interviewed the woman in charge of cooking and feeding the child meals. For consumption pattern, to calculate dietary micronutrients intakes, the database of consumed food items was linked to food composition data based on the following database: the Vietnamese food composition database [12].

### 2.2. Indicators for Assessing Zinc Status of Populations

#### 2.2.1. Dietary Assessment

Inadequate intake of zinc is by far the most likely cause of zinc deficiency for people living in developing countries. This is because most diets in developing countries have a very low bio-availability of zinc, and at the same time poor sanitation and hygiene generate a high prevalence of recurrent infections such as diarrhea that increase the requirements for zinc. Food items which contain plentiful of zinc include meat (especially liver), seafood and eggs, all of which are relatively expensive and thus lacking in many diets. Moreover, although many foods contain zinc at lower levels, the diets in many developing countries also contain a lot of unrefined cereals, resulting in high dietary levels of phytates. Phytate inhibits zinc absorption, especially in molar ratio’s >18 [13]. For assessing zinc intake, standard dietary assessment methods are used, such as weighted food records, recall questionnaires or semiquantitative food frequency questionnaires [1]. As zinc is present in many food items, semi-quantitative food frequency questionnaires might be less accurate, as only a selected number of food items are covered. A major drawback of dietary assessment methods is that although national food composition tables with data of local food items are sometimes (but not always) available, these are often incomplete or inaccurate. Moreover, even though data on zinc content is often present for those food items covered, data on phytate concentrations is almost always lacking. Therefore, it is difficult to accurately estimate zinc absorption from dietary assessments. The current daily recommended nutrient intakes (RNI’s) by FAO and WHO for women of reproductive age are 9.8, 4.9 and 3.0 mg/day for a low, medium and high bioavailable diet respectively [14]. For children between 7 months and 6 years, these figures range from 0.8 mg (for an almost exclusively breast-fed child) to 9.6 mg for a 6 year old child consuming a low bioavailable diet [14]. Interestingly, the RNI for a low bioavailable diet for 7–12 months old child (8.4 mg) is hardly different from a 4–6 years old child (9.6 mg), showing that maintaining adequate zinc intakes during the first 2 years of life might be difficult in developing countries.

#### 2.2.2. Plasma Zinc Concentrations

Plasma (or serum) zinc concentration is the most widely used biomarker to determine zinc status. Plasma zinc concentrations normally respond to zinc supplementation, especially in subjects with a low or moderately low baseline [7]. Depriving subjects of zinc results, in general, in a reduction in plasma zinc concentrations. However, plasma zinc concentrations are affected by many other factors, including inflammation, fasting or eating, pregnancy, oral contraceptive use and diurnal rhythm [7,15]. Inflammation causes a depression of zinc concentrations, whereas fasting leads to higher zinc concentrations. Diurnal variation in zinc concentrations can be as large as 20%. Moreover, conditions that lead to hypoalbuminemia also reduce plasma zinc concentrations, as zinc is bound to albumin in the circulation. Moreover, biological samples can easily be contaminated with zinc from the environment for example from blood tubes, and finally (micro)hemolysis of blood samples will increase plasma zinc concentrations as erythrocytes contain approximately 10x more zinc than plasma. Therefore, careful, quality-controlled blood collection procedures using standardized procedures are essential to obtain reliable data on plasma zinc concentrations, and the interpretation of plasma zinc concentration findings can be a real challenge. Using population distribution curves has been suggested to improve the interpretation of zinc concentrations [1], as individual fluctuations are leveled out, but this also leads to a loss of detail, precision and sensitivity of the indicator, giving only population trends. Current cut-offs for zinc deficiency are <9.9 μmol/L for children <10 years (morning, non-fasting) or <8.7 μmol/L (afternoon, non-fasting) and for non-fasting women of reproductive age (aged 18–49 years; WRA, <10.1 μmol/L (morning) and <9.0 μmol/L (afternoon). Wessells *et al.* suggested a lower cut-off at 7.65 μmol/L however to indicate severe zinc deficiency [16].

#### 2.2.3. Urinary Zinc Concentrations

In normal circumstances, zinc excreted with urine accounts for around 15% of the daily losses. However, when consuming a diet low in zinc, the amount of zinc excreted through urine is reduced by 96% [17]. Therefore, urinary zinc concentrations could be a valuable indicator of zinc status. Indeed Lowe *et al*. reported that zinc intake was related to urinary zinc concentrations [7], and the authors found urinary zinc excretion a useful indicator of zinc status, but only in subjects with a moderate zinc status as baseline. However, currently there is not enough data on urinary zinc (often expressed in relation to urinary creatinine) to make solid recommendations on the validity and usefulness as indicator for zinc status.

#### 2.2.4. Anthropometrical and Stunting Data

Zinc deficiency has been associated with growth faltering and stunting, and indeed a meta-analysis of zinc studies showed that supplementation had a positive effect on length growth, especially in children under 2 years of age, and children stunted at baseline [18]. Hence, following this association, data on growth patterns could be useful to estimate the number of subjects likely to be zinc deficient. The International Zinc Nutrition Consultative Group (IZiNCG) used this method to come to global estimates of zinc deficiency [1]. National stunting prevalences of >20% are considered a public health concern by the World Health Organization, and IZiNG uses the same cut-off to indicate substantial risk for zinc deficiency in a country. However, many factors lead to growth faltering, not only zinc deficiency. For instance inadequate intake of energy and/or protein also lead to growth faltering, and deficiency of other nutrients besides zinc, like magnesium or phosphor, will lead to growth faltering as most prominent feature of deficiency. Moreover, data on length growth and stunting is only available for children, and mainly children under the age of 5 years, precluding the use of growth data as indicator for zinc status for other potential risk groups such as pregnant and lactating women.

### 2.3. Anemia Prevalence

The etiologies of iron deficiency and zinc deficiency have much in common, as both are abundant in the same food items (meat, eggs) and absorption of both is inhibited by phytates. Therefore, the prevalence of anemia could perhaps be used as a proxy indicator of zinc status. This would provide the big advantage that anemia prevalence data available for most countries, is cheap to obtain, and field friendly methods such as the Hemocue exist to perform national surveys. We therefore included anemia and other measures of iron status (ferritin) in the present analyses, keeping in mind of course, anemia is not only due to iron deficiency. Indeed, in some Vietnamese populations, iron deficiency accounts for much less than 50% of the anemia found [19].

#### Ethical Consent

The Scientific Committees of the National Institute of Nutrition (NIN) (Hanoi, Vietnam) and of the Ministry of Health (Hanoi, Vietnam) reviewed and approved the study protocol (204/VDD-QLKH; 25/5/2010). Before enrollment, all women were informed verbally and in writing about the aims and procedures of the study, and written informed consent was obtained from all women and children, via their mother or guardian approval for the later.

## 3. Results

To evaluate for associations or discrepancies between the different indicators of zinc status that are listed above, we examined data on indicators for zinc status from the recent large, nation-wide micronutrient survey in Vietnam described above [8]. In total, 1526 women and 586 children from 1526 households in 19 provinces were surveyed. Roughly half of them lived in an urban area. The different indicators measured in the study to estimate the “prevalence” of zinc deficiency showed very different prevalences of zinc deficiency Table 1.

**Table 1:** Prevalence of low “zinc status” in Vietnamese women of reproductive age (*n* = 1526) and their children aged 6–60 months (*n* = 586).

	Method Used to Assess Zinc Status of the Population				
	Low Plasma Zinc Concentrations	Prevalence of Stunting	Dietary Assessment *	Anemia Prevalence	Iron Deficiency Prevalence
Women	67%	NA	36.0%	12%	14%
Children	52%	23%	50.8%	9%	13%

*: See Laillou *et al*. 2012 [20,21].

Prevalence of low “zinc status” in Vietnamese women of reproductive age (*n* = 1526) and their children aged 6–60 months (*n* = 586) participating in a national wide micronutrient survey in 2010 as defined by low plasma zinc concentration (<9.9 μmol/L for children and <10.1 μmol/L for women), stunting in children (Height for Age *Z*-score < −2), zinc intake below the RDA (RDA of 8 mg/day for women; 3 mg/day for children <3 years and 5 mg/day for children 4–5 years), anemia (<110 g/L for children <5 years of age and <120 g/L for women) and iron deficiency (ferritin < 15 μg/L) (Table 1). 

### 3.1. Public Health Implications

Clearly, these data pose a major public health policy problem, as it would be very difficult to clearly inform policy makers on whether zinc deficiency is a real public health problem in Vietnam or not. Which indicator should be used? If we focus on biochemical evidence such as plasma zinc concentrations, the majority of the Vietnamese population is zinc deficient, clearly a serious public health problem which warrants immediate nutritional interventions. However, if we base our recommendations on anemia or iron deficiency data, the problem is far less acute. The dietary intake data give an estimate for the prevalence of zinc deficiency in between the plasma zinc and anemia/iron data. Interestingly, for children, the data on dietary intake and plasma zinc concentrations appear to correlate to some extent, with approximately half of the population being identified as at risk for zinc deficiency. However, the association is not as strong as it seems from these numbers. There is a lack of consistency between these data, as having a zinc intake below the recommended RDA was not a risk factor for having low plasma zinc concentrations in the children (RR = 1.02, 95% CI 0.82–1.28) and only borderline significant in the women (RR = 1.15, 95% CI 1.00–1.32). Ignoring cut-offs, and examining the data as continuous variables showed that dietary intake data did correlate to plasma zinc concentrations in the women, although the correlation was weak (R = 0.08, *p* = 0.002, linear regression analysis). There was no significant association between dietary intake data and plasma zinc concentrations in the children.

However, if, based on low plasma zinc concentrations, 50% of the children are zinc deficient, why aren’t 50% of the children stunted? The answer of course is that stunting is just a statistically defined point somewhere in the growth curves, whereas growth retardation is a long process seen in most children living in developing countries. It has become clear that the onset of linear growth faltering in South East Asia is probably within a few months of birth, and that the most sensitive period for intervention is prior to 18 months of age. It is also clear that poor zinc status plays a role in this growth retardation, as providing infants with high quality complementary foods including zinc can prevent growth faltering to a large extent [22]. However, whether or not a child falls below the cut-off defined for stunting, has only partly to do with whether a child has a poor zinc status at a given time point. It is the outcome of a long growth trajectory with growth faltering and (incomplete) catch-up growth alternating with many different possible causes, ranging from nutritional deficiency to infectious disease. A measurement at a single time point will therefore not be very informative to understand the reasons for the observed growth deficit. In any case, it is clear that using only stunting prevalence data to identify a population at risk for zinc deficiency is not accurate or sensitive enough. A more reliable method could be to scrutinize growth patterns during childhood, to see whether growth faltering occurs, and at what time point. However, given the fact that growth faltering has a multi-factorial etiology, and growth impairment develops very gradually, growth data can never be more than a rough indicator of zinc status and will not necessarily reflect current zinc status.

### 3.2. Why Is Zinc Different from the Rest?

The difference between zinc and other micronutrients such as iron or vitamin A can be understood more easily through the concept of type I/type II nutrients as developed by M. Golden with zinc being a so-called type II nutrient, whereas iron and vitamin A are type I nutrients [23]. Whereas type I nutrients can be considered as the ‘classic’ physiologic nutrients, type II nutrients can be considered as ‘growth nutrients’ that is, nutrients so essential to growth that in a state of deficiency, (cell) growth will stop. However, as these type II nutrients are present in all tissues, there is no clearly identifiable ‘storage’ compartment from which the nutrient can be mobilized. Deficiency of any one of these nutrients will foremost lead, at least in children, to growth faltering as the primary metabolic reaction to deficiency. If deficiency continues, the human body will switch to catabolism: breakdown of tissue to recycle the lacking type II nutrient in order to safeguard basic essential metabolic processes. A mild deficiency of any type II nutrient will therefore lead to growth faltering, whereas a more severe deficiency will lead to cachexia and wasting [24]. As can be appreciated from this concept, deficiency of any type II nutrient (zinc, magnesium, phosphorus) will lead to growth retardation, making the symptom of growth faltering or stunting a very unspecific indicator for deficiency of any type II nutrient.

Treatment or prevention of deficiency also differs between type I and type II nutrients [25]. For type I nutrients, providing sufficient amounts of the nutrient which was deficient will normally restore the biochemical function. So, in iron-deficiency anemia, providing only iron will normally result in an increase in hemoglobin concentrations and resolve the anemia. In contrast, to counter the growth impairment after a type II nutrient deficiency, one will need to provide all the type II nutrients to enable catch-up growth, as catch-up growth involves anabolism, with the production of new tissue and organ mass, increasing lean body mass, weight, and length, and hence increased requirements for all type II nutrients. For instance, requirements for magnesium and phosphorus (both type II nutrients) for a zinc deficient child receiving zinc treatment are higher than for a normal child of the same age if catch-up growth is required [24].

Again, this has consequences for the assessment of zinc status. Suppose that there is no improvement in growth indices in stunted children after zinc supplementation. Does this mean that zinc deficiency was not the cause of stunting? Or was zinc deficiency the primary reason for the stunting, but is the lack of a beneficial effect after zinc supplementation caused by relative deficiencies of other type II nutrients? Hence, the response to zinc supplementation as indicator for initial zinc deficiency will lack both specificity and sensitivity, as relative deficiency of other type II nutrients can mask the response to zinc supplementation [26].

Another interesting discrepancy in the data from Vietnam is the large difference between the prevalence of iron and zinc deficiency. Iron deficiency, based on plasma ferritin and soluble transferrin receptor concentrations were found in only 13%–14% of the subjects (children and women). This correlated well with the relatively low prevalence of anemia (9%–12%). However, if iron and zinc availability from the diet are similar, how come iron deficiency is not far more common? Indeed, median iron intakes in the women and children were estimated to be respectively 12.8 and 4.8 mg/day [15,16], much below the recommended intakes of 29–58 and 6–13 mg/day respectively, assuming an unrefined diet with 5%–10% bioavailability of the consumed iron. Perhaps the bioavailability of iron has recently improved in the Vietnamese diet, as indeed the consumption of meat and dairy products has increased in the last decade. However, in this case, zinc bioavailability and thereby zinc status should also have increased. Another possibility is that additional iron is provided through another source, for example from drinking water from wells, which are usually not covered in dietary assessments.

Not only does the estimated prevalence between iron and zinc deficiency differ considerably, the correlation or overlap between these indicators is also problematic. This becomes more clear when comparing the deficient subjects as identified by the respective indicators. Recently, Wessells *et al*. [16] proposed a new cut-off for severe zinc deficiency, using a plasma zinc concentration of 7.65 μmol/L (50 μg/dL). Using this cut-off, 13% of the children and 17% of the WRA would be classified as having severe zinc deficiency. This appears at first sight to be more in line with the prevalence of iron deficiency. However, there was no correlation between having low ferritin concentrations (<15 μg/L or <30 μg/L) and having low zinc concentrations (<7.65 μmol/L) both in the children and the women (Pearson chi-square *p* > 0.30 for all). Hence, we need to conclude that the use of anemia or iron deficiency status appears not to be a suitable proxy indicator for zinc status on population level.

Based on both the dietary intake and plasma zinc data from this nation-wide survey [8] as well as some earlier smaller studies using plasma zinc as biomarker for zinc status [27], the National Institute of Nutrition, Ministry of Health, Vietnam now considers zinc deficiency a public health concern in Vietnam, and is investigating tools such as food fortification to improve zinc status of the general population [28]. Unfortunately, at present it is unclear what impact can be expected from for example nation-wide mandatory rice fortification with zinc, as it is unclear how a food product fortified with zinc affects zinc status, or how it affects plasma zinc concentrations (and as discussed earlier, how plasma zinc concentrations are related to zinc status). Indeed, a recent study from Senegal showed that porridge fortified with 6 mg of zinc did not lead to an increase in plasma zinc concentrations, whereas the same amount of zinc as a liquid supplement did [29]. Moreover, without a clear expected impact on plasma zinc concentrations, it will be difficult to design a monitoring and evaluation plan for the intervention. Policy makers should be informed in advance of these issues, to prevent disappointment when monitoring progress in program indicators, with some outcome indicators not showing any effect while the program might be effective in itself. A possible approach here is to specifically include long-term outcomes that reflect the broader impact of improving zinc nutrition such as growth (for example stunting reduction, birth weight) and health (for example infectious disease morbidity or mortality).

## 4. Conclusions

To date, there is no consensus on which indicators are best to use for the determination of zinc status of a population. This has led to many different indicators being used, making comparison among surveys and studies difficult, and has led to confusion about the extent of zinc deficiency in the world. Although indirect indicators such as stunting or iron deficiency prevalence have been used, these are imprecise and may lead to erroneous estimates of zinc deficiency. Dietary intake data might provide a rough indication of zinc intake, but availability of adequate food composition tables in many developing countries limit their use. More direct indicators of zinc status such as plasma and urinary zinc concentrations are probably the best indicators currently available, and these should be used if data is available to estimate the prevalence of zinc deficiency in a population. Long-term effects of zinc nutrition such as on growth and health could be useful to evaluate interventions in a meaningful way on a population level.

To gain better insight in the sensitivity and specificity of some of the biomarkers for zinc status mentioned above such as plasma zinc or urinary zinc, more research is needed, linking these indicators to functional outcomes (diarrhea incidence; growth faltering) on one hand, and unequivocal indicators of total body zinc content (by use of stable isotopes) on the other hand. Ideally, these studies should be done over a wide range of conditions such as subjects with marginal and adequate zinc status, and subjects with inflammation. Moreover, the impact of selected interventions such as fortification of staple foods with zinc could be evaluated with stable isotopes in combination with several other biomarkers to determine which biomarker is most suited to be used for program evaluation purposes. Unfortunately, the use of stable isotopes is too expensive to be used on a routine basis. At the moment, the real extent of the prevalence of zinc deficiency in the world is unknown, and we have nothing more than a very rough estimate not only of the prevalence, but also of the potential impact on global health that can be expected from improving zinc nutrition in populations.

## References

[1] Hotz C., Brown K.H. (2004). Assessment of the Risk of Zinc Deficiency in Populations and Options for its Control. IZINCG Technical Document #1.

[2] Prasad A.S., Halsted J.A., Nadimi M. (1961). Syndrome of iron deficiency, anemia, hepatosplenomegaly, hypogonadism, dwarfism, and geophagia. Am. J. Med..

[3] Hambidge M. (2000). Human zinc deficiency. J. Nutr..

[4] Black R.E., Victora C.G., Walker S.P., Bhutta Z.A., Christian P., de Onis M., Ezzati M., Grantham-McGregor S., Katz J., Martorell R. (2013). Maternal and child undernutrition and overweight in low-income and middle-income countries. Lancet.

[5] WHO (1996). Indicators for Assessing Vitamin A Deficiency and Their Application in Monitoring and Evaluating Intervention Programmes.

[6] Mei Z., Cogswell M.E., Parvanta I., Lynch S., Beard J.L., Stoltzfus R.J., Grummer-Strawn L.M. (2005). Hemoglobin and ferritin are currently the most efficient indicators of population response to iron interventions: An analysis of nine randomized controlled trials. J. Nutr..

[7] Lowe N.M., Fekete K., Decsi T. (2009). Methods of assessment of zinc status in humans: A systematic review. Am. J. Clin. Nutr..

[8] Laillou A., Pham T.V., Tran N.T., Le H.T., Wieringa F., Rohner F., Fortin S., Le M.B., Tran do T., Moench-Pfanner R. (2012). Micronutrient deficits are still public health issues among women and young children in Vietnam. PLoS ONE.

[9] De Onis M., Onyango A.W., Borghi E., Garza C., Yang H. (2006). Comparison of the World Health Organization (WHO) Child Growth Standards and the National Center for Health Statistics/WHO international growth reference: Implications for child health programmes. Public Health Nutr..

[10] Tan K.C.B. (2004). Appropriate body-mass index for Asian populations and its implications for policy and intervention strategies. Lancet.

[11] Dop M.C., Le B.M., Laillou A., Sénémaud B., Nguyen C.K., Khoi H.H. Validation of the 24-h recall against the weighing method at household level-advantage of comparing methods on the same and separate days: A case study from Vietnam. Proceedings of the Fourth International Conference on Dietary Assessment Methods.

[12] Ministry of Public Health and National Institute of Nutrition (2000). Nutritive Composition Table of Vietnamese Food.

[13] Gibson R.S., Bailey K.B., Gibbs M., Ferguson E.L. (2010). A review of phytate, iron, zinc, and calcium concentrations in plant-based complementary foods used in low-income countries and implications for bioavailability. Food Nutr. Bull..

[14] WHO/FAO (2004). Vitamin and Mineral Requirements in Human Nutrition.

[15] Wieringa F.T., Dijkhuizen M.A., West C.E., Northrop-Clewes C.A. (2002). Estimation of the effect of the acute phase response on indicators of micronutrient status in Indonesian infants. J. Nutr..

[16] Wessells K.R., King J.C., Brown K.H. (2014). Development of a plasma zinc concentration cutoff to identify individuals with severe zinc deficiency based on results from adults undergoing experimental severe dietary zinc restriction and individuals with acrodermatitis enteropathica. J. Nutr..

[17] King J.C., Shames D.M., Lowe N.M., Woodhouse L.R., Sutherland B., Abrams S.A., Turnlund J.R., Jackson M.J. (2001). Effect of acute zinc depletion on zinc homeostasis and plasma zinc kinetics in men. Am. J. Clin. Nutr..

[18] Brown K.H., Peerson J.M., Rivera J., Allen L.H. (2002). Effect of supplemental zinc on the growth and serum zinc concentrations of prepubertal children: A meta-analysis of randomized controlled trials. Am. J. Clin. Nutr..

[19] Nga T.T., Winichagoon P., Dijkhuizen M.A., Khan N.C., Wasantwisut E., Furr H., Wieringa F.T. (2009). Multi-micronutrient-fortified biscuits decreased prevalence of anemia and improved micronutrient status and effectiveness of deworming in rural Vietnamese school children. J. Nutr..

[20] Laillou A., Le B.M., Le T.H., Khan N.C., Panagides D., Wieringa F., Berger J., Moench-Pfanner R. (2012). An assessment of the impact of fortification of staples and condiments on micronutrient intake in young vietnamese children. Nutrients.

[21] Laillou A., Berger J., Le B.M., Pham V.T., Le T.H., Nguyen C.K., Panagides D., Rohner F., Wieringa F., Moench-Pfanner R. (2012). Improvement of the Vietnamese diet for women of reproductive age by micronutrient fortification of staples foods and condiments. PLoS ONE.

[22] Pham V.P., Nguyen V.H., Salvignol B., Treche S., Wieringa F.T., Dijkhuizen M.A., Nguyen C.K., Pham D.T., Schwartz H., Berger J. (2012). A six-month intervention with two different types of micronutrient-fortified complementary foods had distinct short- and long-term effects on linear and ponderal growth of Vietnamese infants. J. Nutr..

[23] Golden M.H.N., Guandalini S. (2004). Malnutrition. Textbook of Pediatric Gastroenterology and Nutrition.

[24] Golden M.H. (2009). Proposed recommended nutrient densities for moderately malnourished children. Food Nutr. Bull..

[25] Wieringa F.T., Dijkhuizen M.A., Berger J. (2013). Consequences of micronutrient deficiency and interventions to improve micronutrient status. Nutrition in Infancy.

[26] Piwoz E., Sundberg S., Rooke J. (2012). Promoting healthy growth: What are the priorities for research and action?. Adv. Nutr..

[27] Van Nhien N., Khan N.C., Ninh N.X., van Huan P., Hop le T., Lam N.T., Ota F., Yabutani T., Hoa V.Q., Motonaka J. (2008). Micronutrient deficiencies and anemia among preschool children in rural Vietnam. Asia Pac. J. Clin. Nutr..

[28] Khanh Van T., Burja K., Thuy Nga T., Kong K., Berger J., Gardner M., Dijkhuizen M.A., Hop le T., Tuyen le D., Wieringa F.T. (2014). Organoleptic qualities and acceptability of fortified rice in two Southeast Asian countries. Ann. N.Y. Acad. Sci..

[29] Lo N.B., Aaron G.J., Hess S.Y., Dossou N.I., Guiro A.T., Wade S., Brown K.H. (2011). Plasma zinc concentration responds to short-term zinc supplementation, but not zinc fortification, in young children in Senegal1. Am. J. Clin. Nutr..

